# Preparation and Characterization of CCTO/PDMS Dielectric Elastomers with High Dielectric Constant and Low Dielectric Loss

**DOI:** 10.3390/polym13071075

**Published:** 2021-03-29

**Authors:** Wenqi Wang, Guanguan Ren, Ming Zhou, Wei Deng

**Affiliations:** School of Materials Science and Engineering, Harbin University of Science and Technology, No. 52 Xuefu Road, Nangang District, Harbin 150040, China; 1820200021@stu.hrbust.edu.cn (W.W.); 1820200010@stu.hrbust.edu.cn (G.R.); 1820200019@stu.hrbust.edu.cn (M.Z.)

**Keywords:** dielectric elastomer, silicone rubber, copper calcium titanate, electrodeformation

## Abstract

Dielectric elastomer (DE) is a type of electric field type electroactive polymer material that can produce greater deformation under the action of an electric field and has a faster recovery speed. It has the advantages of high energy density, large strain, low quality, and commercialization, and has become the most widely concerned and researched electroactive polymer material. In this study, copper calcium titanate (CCTO) particles with a large dielectric constant were selected as the filling phase, and a silicone rubber (PDMS) with better biocompatibility and lower elastic modulus was used as the matrix to prepare CCTO/PDMS, which is a new type of dielectric elastomer material. The structure of the dielectric elastomer is analyzed, and its mechanical properties, dielectric properties, and driving deformation are tested. Then, KH550, KH560, and KH570 modified CCTO is used in order to improve the dispersibility of CCTO in PDMS, and modified particles with the best dispersion effect are selected to prepare dielectric elastomer materials. In addition, mechanical properties, dielectric properties, and driving deformation are tested and compared with the dielectric elastomer material before modification. The results show that as the content of CCTO increases, the dielectric constant and elastic modulus of the dielectric elastomer also increase, and the dielectric loss remains basically unchanged at a frequency of 100 Hz. When the filling amount reaches 20 wt%, the dielectric constant of the CCTO/PDMS dielectric elastomer reaches 5.8 (100 Hz), an increase of 120%, while the dielectric loss at this time is only 0.0038 and the elastic modulus is only 0.54 MPa. When the filling amount is 5 wt%, the dielectric elastomer has the largest driving deformation amount, reaching 33.8%. Three silane coupling agents have been successfully grafted onto the surface of CCTO particles, and the KH560 modified CCTO has the best dispersibility in the PDMS matrix. Based on this, a modified CCTO/PDMS dielectric elastomer was prepared. The results show that the improvement of dispersibility improves the dielectric constant. Compared with the unmodified PDMS, when the filling content is 20 wt%, the dielectric constant reaches 6.5 (100 Hz). Compared with PDMS, it has increased by 150%. However, the improvement of dispersion has a greater increase in the elastic modulus, resulting in a decrease in its strain parameters compared with CCTO/PDMS dielectric elastomers, and the electromechanical conversion efficiency has not been significantly improved. When the filling amount of modified CCTO particles is 5 wt%, the dielectric elastomer has the largest driving deformation, reaching 27.4%.

## 1. Introduction

Among the various new materials, polymer smart materials have attracted widespread attention from the academic community due to their advantages of light weight, high strength, large deformation, good biocompatibility, good processing performance, and low price [1,2]. Polymer smart materials can produce corresponding stress responses to specific external stimuli, such as electric field changes, temperature changes [3], light [4], magnetism [5], pH, and other stimuli that produce specific responses, including structural changes, color changes [6], and shape changes, as well as other forms. Therefore, it has played a good role in promoting the interdisciplinary development and cross development of other disciplines.

The deformation scale of DE material under the action of electric field is very large, the area deformation can generally exceed 100%, and its response to stimuli is also very sensitive. The response speed is usually in the millisecond level, and the maximum stress can reach 3.3 above MPa, the energy density reaches 3.4 J/g, and the electromechanical conversion efficiency is above 80% [7]. In other words, the deformation scale and efficiency of DE materials are very close to those of electro-deformable materials, but they require lower energy, lighter weight, and overall performance similar to the muscle tissue. In addition, these data are constantly on the rise, thus research on DE materials is very necessary [8,9].

**Figure 1:**
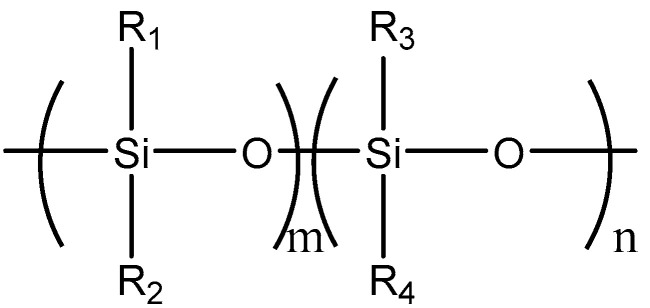
Molecular formula of silicone rubber material.

Silicone rubber is not sensitive to temperature and humidity, and at the same time, it has a wide temperature range and a very fast response speed. In addition, silicone rubber materials have good biocompatibility and are the first choice for artificial muscle materials, which are widely used in the medical field [10,11,12]. The molecular formula of silicone rubber is shown in Figure 1:

Commonly used silicone rubber materials are the HS3 series rubber, Sylgard series silicone rubber, CF series silicone rubber, etc. [10]. However, as a common non-polar rubber, silicone rubber has a low dielectric constant and a higher elastic modulus than VHB (a series of polyacrylate pressure-sensitive adhesives produced by 3M in the United States) series materials. Therefore, a larger field strength is required to obtain a larger amount of deformation. When the amount of deformation is large enough, due to the inherent characteristics of rubber, its elastic modulus will also increase rapidly, which further limits its development [13,14,15].

The most common way to improve the performance of DE is to increase the dielectric constant of DE, which is also an active field of DE material research. Increasing the dielectric constant of the material can effectively reduce the driving voltage and achieve higher electromechanical conversion efficiency. Common methods include filling ferroelectric ceramic materials and filling conductive fillers. Kumar et al. [16] prepared BT randomly filled PDMS and BT oriented filled PDMS dielectric elastomers. Compared with silicone composites cured in the absence of an electric field, they are filled with 15% filler content in the presence of an electric field. The cured silicone composite material has a higher dielectric constant (3.8, 100 Hz). The elongation at break of the oriented particle composite material increases, and when the filling load is 15%, the actuation strain area increases from 5.5% to 18%. By adding 5.1 vol% CCTO to PDMS, Romasanta et al. [17] significantly improved the dielectric and mechanical properties of the dielectric elastomer of the CCTO/PDMS composite material. The maximum deformation of the dielectric elastomer increased by 100%. Compared with PDMS, the driving voltage required to achieve the same deformation is reduced by 25%. Gall et al. [18] explored the electrical performance of PMN-PT/PMDS composites. With a 30 wt% addition of composites, the dielectric constant increased from 8 to 32 at 10 Hz, but the loss and elastic modulus also increased. Galantini et al. [19] doped PU with functionalized multi-walled carbon nanotubes to significantly increase the dielectric constant and reduce the elastic modulus.

Copper calcium titanate (CCTO), as a functional ceramic material, has many excellent properties. The most typical advantage is its excellent dielectric properties. It is a giant dielectric ceramic material with a large relative dielectric constant (under the condition of 1 kHz, the measured dielectric constant is about 1500), and its relative dielectric constant is very stable (DC-10^6^ Hz). When the frequency changes greatly or the temperature changes (100–400 K), the relative permittivity changes are very small [20,21].

In this study, the giant dielectric ceramic particles of copper calcium titanate (CCTO) were used as a reinforcing material to be dispersed in PDMS, in order to obtain a dielectric elastomer with high dielectric constant and low elastic modulus, using silane coupling modified CCTO to enhance its dispersibility in PDMS. On the other hand, the influence of the filler content on the properties of dielectric elastomers was explored.

## 2. Experimental

### 2.1. Materials

Analytical pure absolute ethanol, tetrahydrofuran, Yaohua brand silane coupling agents KH550(NH_2_(CH_2_)_3_Si(OC_2_H_5_)_3_), KH560(CH_2_CH(O)CH_2_O(CH_2_)_3_Si(OCH_3_)_3_), and KH570(CH_2_=C(CH_3_)COO(CH_2_)_3_Si(OCH_3_)_3_), Dow Corning′s Sylgard 184 silicone rubber and its matching curing agent, as well as industrial pure copper calcium titanate particles were produced by the lock screen of Hunan Kelai New Material Co., Ltd (Changde, China).

### 2.2. Measurements

A FEI Sirion200 field emission scanning electron microscope was used to characterize the microstructure of the particles. The unmodified CCTO particles and @CCTO modified with a silane coupling agent were observed, the energy spectrum was drawn, and the dispersion in PDMS was characterized. The unmodified CCTO particles and @CCTO modified with a silane coupling agent were tested and characterized by an X-ray diffractometer produced by the Dutch company PANalytical, and the crystal form changes and the interlamellar spacing were analyzed. A Nicolet iS10 infrared spectrometer was used to test and characterize the unmodified CCTO particles and @CCTO modified with a silane coupling agent, and then the unmodified and modified PDMS films were tested. The Alpha-A broadband dielectric spectrometer produced by Novocontrol in Germany was used to test the dielectric properties of the composite material. The AGS-J10 electronic universal testing machine from Shimadzu Corporation of Japan was used to test the elastic modulus of the composite material. The DTZG-60 high-voltage DC power supply was used to test the electro-deformation behavior of the samples.

### 2.3. Preparation of CCTO/PDMS Dielectric Elastomer

First, the CCTO was placed in a vacuum oven and taken out after a constant weight. A certain amount of CCTO was weighed and grinded with an agate mortar for 20 min until there were no large particles. Then, the solution was added to tetrahydrofuran, sealed, and ultrasonically dispersed for 20 min. After the dispersion was uniform, the CCTO tetrahydrofuran solution was mixed with the A and B components of Sylgard 184 silicone rubber mixed in a 20:1 mass ratio, and stirred well for 30 min to obtain a uniform. The mixed solution was poured into the preheated self-made iron mold coated with a white petrolatum release agent, and heated in an oven at 80 °C for 2 h. After the curing was complete, the sample was taken out to obtain the CCTO/PDMS composite material.

### 2.4. Preparation of CCTO/PDMS Dielectric Elastomers Modified by Different Silane Coupling Agents

A certain amount of dried CCTO was added to absolute ethanol and dispersed for 30 min. In another beaker, 1% KH550 of CCTO mass (KH560 and KH570 are the same) and absolute ethanol were added and dispersed for 30 min. Then, the dispersed CCTO ethanol solution and the silane coupling agent ethanol solution were mixed, stirred for 2 h, and after the mixture fully reflected, ethanol was used to clean, centrifuge, and remove the supernatant three times. After that, the solution was placed in a 70 °C oven, baked to a constant weight, and taken out to obtain a silane coupling agent modified CCTO, which was marked as @CCTO. The preparation method of @CCTO/PDMS was the same as that of CCTO/PDMS.

### 2.5. Manufacturing of Dielectric Elastomer Actuators

The DE film was made into a circular sheet with a radius of 55 mm, and the edge of the circular sheet was fixed with a self-made PMMA clamp. A conductive carbon paste with a radius of 10 mm and a thickness of 1 mm was applied evenly on the centers of both sides of the DE film, and connected to the high-voltage power supply with a wire. During the boosting process, a camera was used to record the deformation. In the Photoshop software, the number of pixels represents the area change. Three elastomers for each group of samples were prepared and the average value was taken.

## 3. Results and Discussion

### 3.1. Characterization of CCTO Particle Microstructure

**Figure 2:**
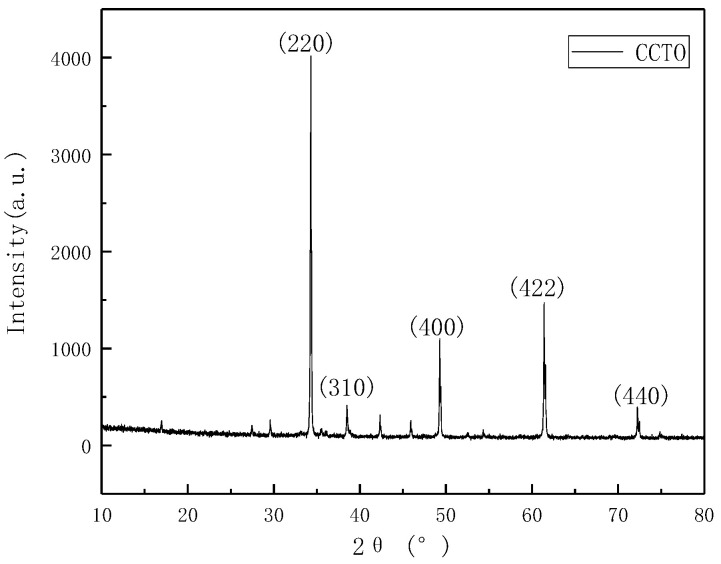
XRD spectrum of unmodified copper calcium titanate (CCTO).

As observed in Figure 2, the main features of the calcium copper titanate are consistent with the position and intensity of the diffraction peaks of the standard spectrum of copper calcium titanate (JCPD Cade No. 21-0140). In addition, there are fewer and smaller impurity peaks, indicating that the purity of copper calcium titanate is relatively high and the crystal form is uniform. The stronger main peak intensity of CCTO means that its crystallinity is higher and the crystal grains are larger.

**Figure 3:**
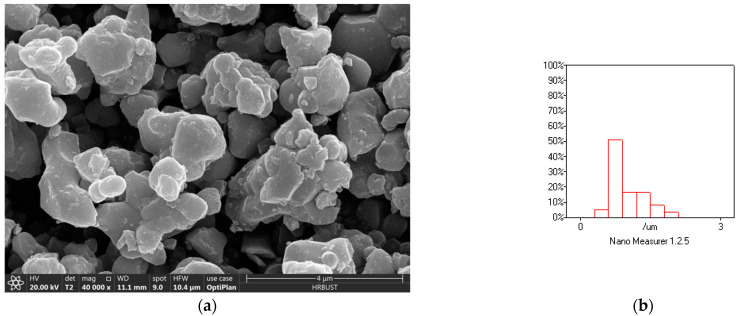
(**a**) Particle morphology of CCTO; (**b**) particle size distribution of CCTO.

The SEM analysis result of CCTO particle morphology is shown in Figure 3a. As can be seen, the CCTO particles are irregularly spherical, and have uneven particle sizes. The particle size distribution diagram is shown in Figure 3b, with an average particle size of 1.2 µm.

### 3.2. Analysis of Microscopic Appearance of Three Modified Particles

**Figure 4:**
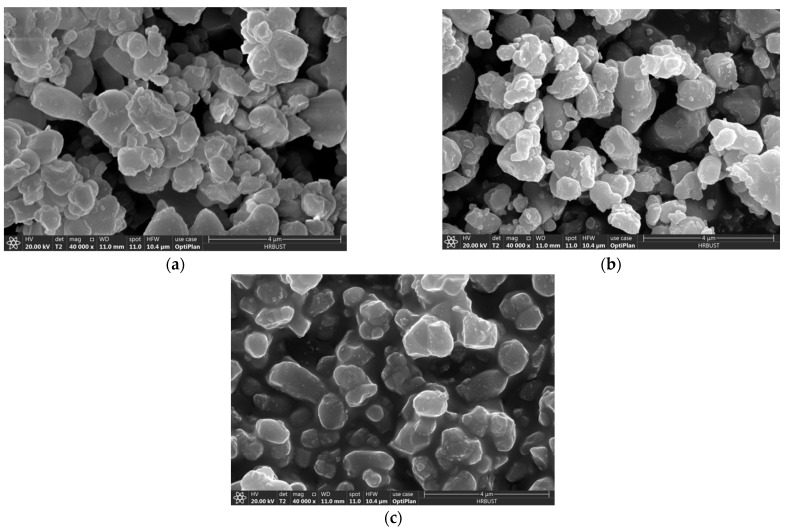
SEM of CCTO particles modified by the silane coupling agent: (**a**) KH550 modified CCTO; (**b**) KH560 modified CCTO; (**c**) KH570 modified CCTO.

Figure 4 shows the SEM image of three kinds of modified particles. Observing the spectrum shows that the addition of the coupling agent does not make it agglomerate. The dispersion is still very good, and the particle size has not changed significantly. Table 1 shows the energy spectrum comparison table of unmodified CCTO and modified CCTO. Using the energy spectrum analysis, it can be seen that there are C and Si elements in the system, indicating that after full washing, modifier elements are still found in the system and the silane coupling agent successfully reacted on the particle surface.

**Figure 5:**
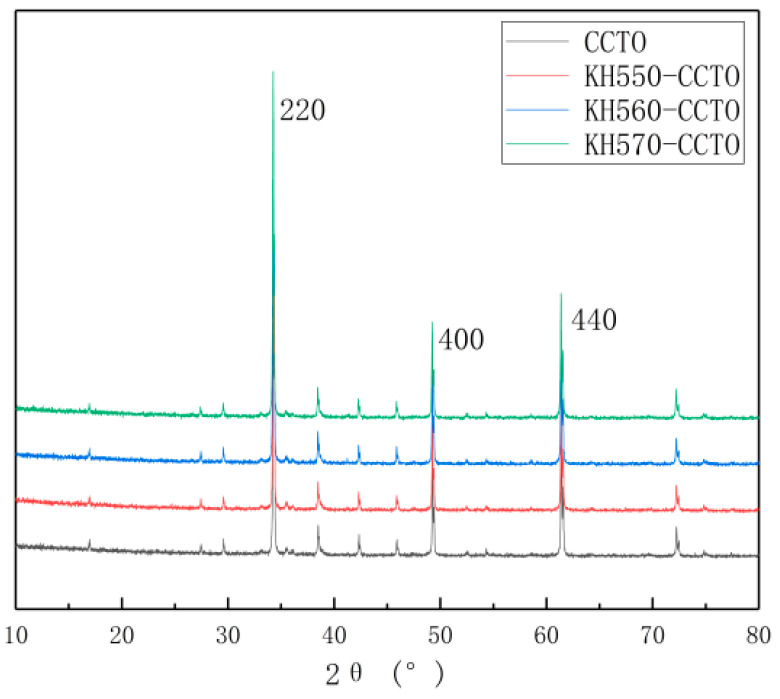
XRD spectrum of unmodified and modified CCTO.

Figure 5 shows the XRD spectra of the unmodified CCTO and modified CCTO. As can be seen, the peak position has not changed significantly, indicating that the addition of the silane coupling agent does not affect the crystal structure, only the adhesion on the surface of the crystal.

**Figure 6:**
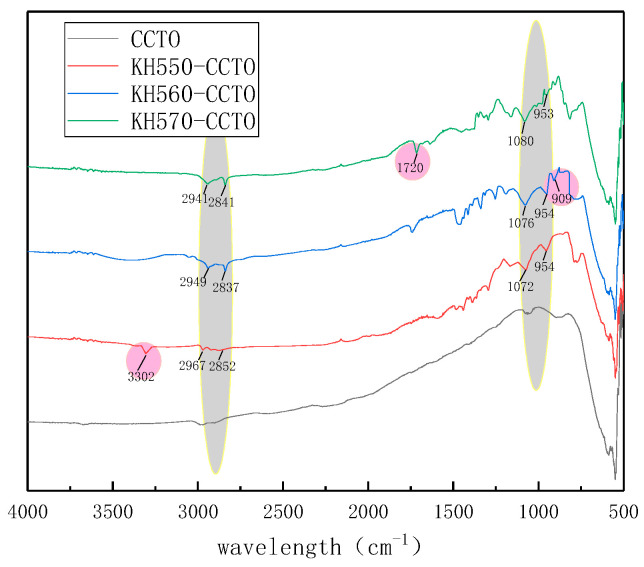
Infrared spectra of unmodified CCTO and modified CCTO.

**Figure 7:**
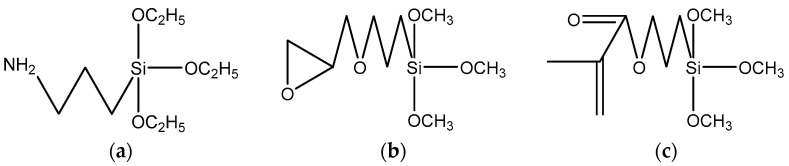
(**a**) Simplified structure of KH550; (**b**) simplified structure of KH560; (**c**) simplified structure of KH570.

Figure 6 shows the IR comparison of unmodified CCTO and modified CCTO. The simplified structure of the three silane Euro links is shown in Figure 7. On the KH550 modified CCTO, a different infrared absorption peak at 3302 cm^−1^ can be clearly seen, which is the stretching vibration of amino NH peak, thus we can know the presence of amino groups in the system. An infrared absorption peak at 909 cm^−1^ can be seen on the KH560 modified CCTO, which is a characteristic peak of epoxy groups, indicating that there are epoxy groups in the system. On the KH570 modified CCTO, it can be seen that there are different absorption peaks at 1720 and 1631 cm^−1^, which are the absorption peak of the ester carbonyl group and the vibration absorption peak of the carbon-carbon double bond, indicating that the system contains esters, radicals, and carbon-carbon double bonds. The three modified infrared spectra all have absorption peaks at 1080 and 950 cm^−1^, which are the vibration absorption peaks of Si-O bond and Ti-O bond, respectively, indicating that the silane coupling agent and CCTO reacted and successfully grafted to the surface of the particles. Near 2841 and 2941 cm^−1^ are the symmetrical and antisymmetric vibration absorption peaks of —CH_3_ and —CH_2_—, respectively, which come from the chain of the silane coupling agent [22,23,24,25].

### 3.3. Comparison of the Dispersion of CCTO in PDMS before and after Modification

**Figure 8:**
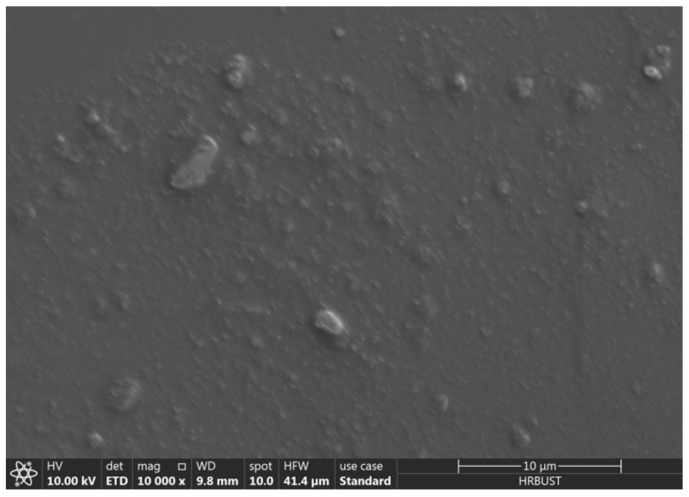
Sectional SEM image of 5 wt% CCTO/ silicone rubber (PDMS).

SEM was used to observe the brittle section of PDMS filled with CCTO, and the results are shown in Figure 8. As can be seen, the dispersion of CCTO in PDMS is generally good, with agglomeration in some areas. However, the overall distribution is uniform, and the end surface is very smooth, which indicates that CCTO and PDMS have good compatibility.

**Figure 9:**
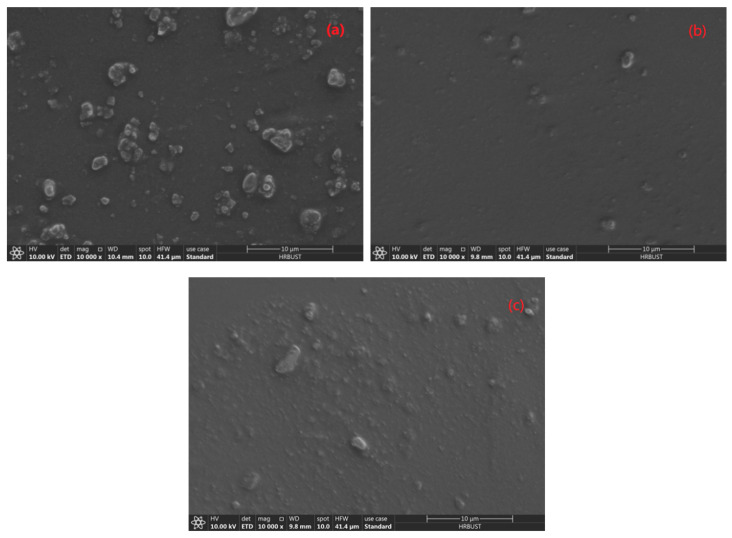
Cross-sectional SEM photos of PDMS composites doped with different silane coupling agents modified CCTO particles: (**a**) KH550-CCTO/PDMS; (**b**) KH560-CCTO/PDMS; (**c**) KH570-CCTO/PDMS.

Figure 9 shows an SEM image of three types of 5 wt% CCTO particles after PDMS dispersion. As can be clearly seen, the particles modified with KH560 have significantly less agglomerates in the matrix than the other two, and the dispersibility is better. Therefore, in the next experiment, the modifier used is KH560, and the CCTO particles modified by KH560 are marked as @CCTO.

### 3.4. Mechanical Properties of Dielectric Elastomer Composites

**Figure 10:**
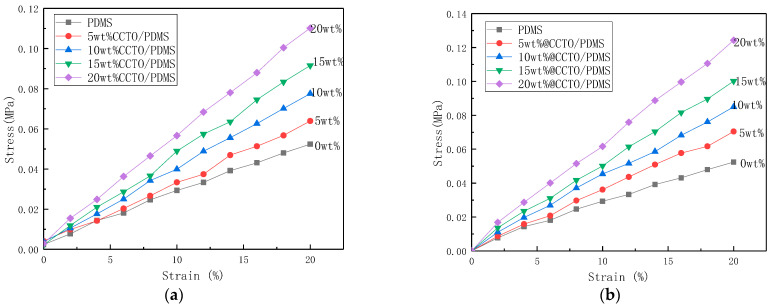
(**a**) The stress-strain curve of CCTO/PDMS composite; (**b**) The stress-strain curve of modified CCTO/PDMS(@CCTO/PDMS) composite.

Figure 10 shows the stress-strain curves of CCTO/PDMS and @CCTO/PDMS composites. By calculating the slope of the curve, the elastic modulus of the material can be obtained.In this paper, the Sylgard 184 silicone rubber is used with a ratio of 20:1 to the curing agent. It has better flexibility and has an elastic modulus of only 0.25 MPa, which greatly improves the electro-induced deformation of the dielectric elastomer. For CCTO/PDMS composites with filling amounts of 5, 10, 15, and 20 wt%, the elastic modulus is 0.3, 0.37, 0.44, and 0.54 MPa, respectively, and the elastic modulus has increased by 20%, 48%, 76%, 116%, respectively. This is due to the fact that the addition of CCTO forms physical crosslinking points with PDMS, and the elastic modulus of inorganic fillers is much higher than that of polymer materials, thus the modulus of elasticity increases. When the filling value is 20 wt%, the elastic modulus is only 0.54 MPa. Although there is a large increase, it still has a lower elastic modulus than the other polymer materials. The low modulus ensures that the DE material has a better strain capacity and can produce greater deformation under the action of an electric field [26].

When the @CCTO filling amount is 5, 10, 15, and 20 wt%, the elastic modulus of @CCTO/PDMS composites are 0.33, 0.41, 0.49, and 0.6 MPa, respectively. Compared with the modified PDMS, it has increased by 32%, 64%, 96%, and 140%, respectively. It can be found that after the KH560 modification, it is higher than the unmodified CCTO filled composite material at the same content. This is due to the fact that the addition of KH560 makes the dispersion of CCTO better, and the interface bonding force is stronger, which increases the Interface effect, thus the elastic modulus rises more obviously.

### 3.5. Dielectric Properties of Dielectric Elastomer Composites

**Figure 11:**
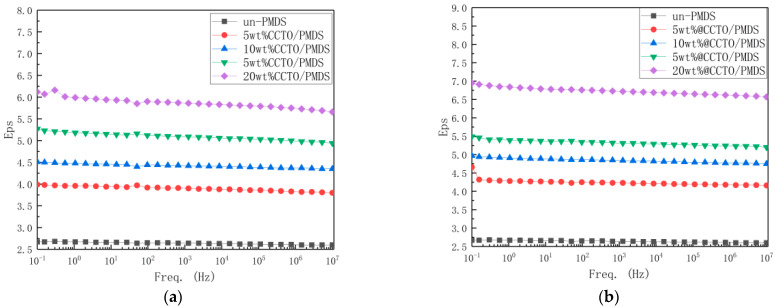
(**a**) The broadband dielectric spectroscopy of CCTO/PDMS composite; (**b**) The broadband dielectric spectroscopy of modified CCTO/PDMS composite.

Figure 11 shows the dielectric constant diagram of different contents of the CCTO/PDMS composite at different frequencies. Since CCTO and PDMS are less dependent on frequency, there is no obvious rise and fall on the curve, and they basically tend to be stable. At 100 Hz, the dielectric constant of the unmodified PDMS is 2.6, and the CCTO/PDMS composites with filling amounts of 5, 10, 15, and 20 wt%. At 100 Hz, the dielectric constants are 3.9, 4.4, 5, and 5.8, with an increase of 50%, 69%, 92%, and 120%, respectively. This is due to the fact that CCTO is a material with a huge dielectric constant. A strong polarization is formed on the interface between the CCTO particles and the PDMS matrix, so the dielectric constant changes very obviously. As the amount of filler increases, the distance between the CCTO particles gets closer, and dipole polarization occurs between the particles, which causes the dielectric constant to increase rapidly. On the other hand, due to the increase of CCTO particles, the interface formed between the CCTO particles and the PDMS matrix also increases, the effect of interface polarization is enhanced, and the dielectric constant of the composite material is further increased [26,27,28].

When the filling amount is 5, 10, 15, and 20 wt%, @CCTO/PDMS composite materials have a dielectric constant of 4.2, 4.8, 5.3, and 6.5 at 100 Hz, respectively, which increase by 62%, 84%, 104%, 150%, respectively. Compared with the unmodified CCTO, both have increased. This is due to the fact that the modified CCTO has enhanced dispersibility, in which the interface becomes more and stronger. The increased interface effect and the more uniform dispersion makes the internal dipolar polarization increase, thus the dielectric constant is higher.

**Figure 12:**
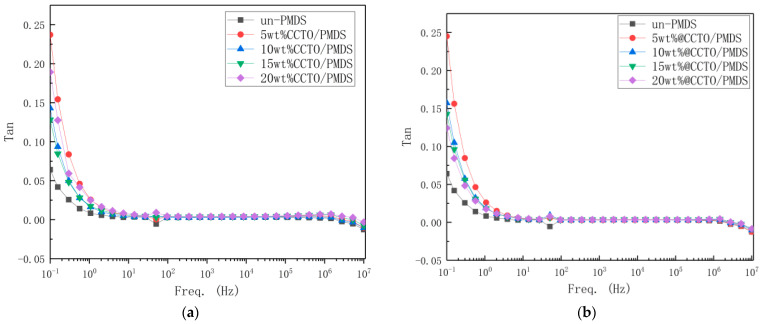
(**a**) The dielectric loss spectrum of CCTO/PDMS composite; (**b**) The dielectric loss spectrum of modified CCTO/PDMS composite.

Figure 12 shows a graph of the change in dielectric loss of the DE material. As can be seen, the dielectric loss of the five materials is consistent above 100 Hz, and all are less than 10^−2^, maintaining a low dielectric loss. Here, the XRD pattern that the crystal forms of CCTO is the same and there are fewer defects. Therefore, as the filling amount increases, the dielectric loss is not significantly affected.

As can be seen, after the KH560 modification, the dielectric loss has not increased, and is still less than 10^−2^, maintaining a low dielectric loss. Except for the 10 wt% @CCTO/PDMS dielectric loss, which is larger than the unmodified dielectric loss, the others are all smaller than before the modification. This situation may be due to experimental errors. As the dispersibility becomes better, the friction between the crystal grains is relatively reduced, the density of the material is increased, and the defects are reduced, thus the dielectric loss also decreases [17].

In the dielectric loss graph, we found that there is a fluctuation when it is lower than 100 Hz. This is due to the fact that the dielectric loss test is a wavelet analysis and is susceptible to external interference. At 100 Hz, the electrodes, wires, and a macroscopic capacitance is formed between the power supplies, which interferes with the signal.

**Figure 13:**
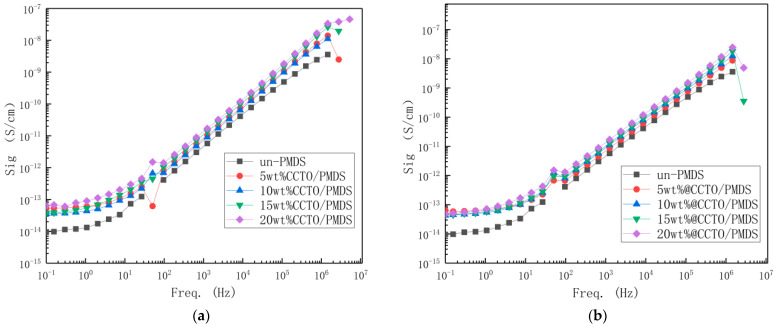
(**a**) Conductivity of CCTO/PDMS composites at different frequencies; (**b**) Conductivity of @CCTO/PDMS composites at different frequencies.

Figure 13 shows the conductivity change diagram of each group of DE materials. Since the dispersion of CCTO in PDMS is relatively uniform, there is no significant change in the curve. Due to the addition of the reinforcement, it is easier for the electrons to pass through the PDMS gap, resulting in a slight increase in its electrical conductivity. However, it is still lower than 10^−7^, which maintains a good insulation performance.

### 3.6. Electro-Deformation Properties of Dielectric Elastomer Composites

**Figure 14:**
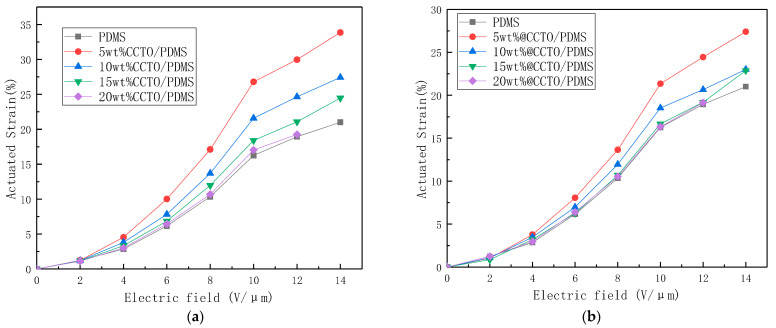
Electrostrain diagram of PDMS and different contents of CCTO (**a**) and @CCTO (**b**) filled PDMS composites.

Figure 14 shows the electrostrain graph of PDMS and different contents of CCTO and @CCTO filled PDMS composites. As shown, when the unmodified CCTO is used to fill PDMS, the electro-deformation of all the materials increases with the increase of the electric field strength, and the electro-deformation of 5 wt% CCTO/PDMS is the highest, reaching 33.8%. The amount of electro-induced deformation decreased with the increase of the filling volume, but they were all higher than that of the unmodified PDMS. After modification, the amount of electro-induced deformation increased, but it was less than the amount of unmodified electro-induced deformation. The maximum deformation is 5 wt% @CCTO/PDMS, which reaches 27.4% at a field strength of 14 V/µm.

In fact, the DE model is also a relatively active field. For example, Wissler et al. [29] started with the structure of dielectric elastomers and gave the Maxwell model. Goulbourne [13], Dorfmann, among others [30] have made improvements based on the source of internal stress after deformation and the nonlinear factors in the deformation process. The essence of these theories is to combine the theory of superelastic large deformation with the theory of electrostatics. According to the thermodynamic theory, Sou et al. [31] obtained the constitutive equations of DE using the conversion process of electrostatic energy and elastic strain energy. More scientific researchers continue to invest in improving these models to make their simulations in different situations and fields more realistic. For example, Bustamante proposed a system of equations suitable for boundary values [32]. Mehenrt et al. [33] proposed a system of constitutive equations for DE materials without pretension based on a finite-strain viscoelastic material model. Additionally, Hossain et al. [34] proposed a large-strain framework based on phenomenology. Putting forward the constitutive equation, these models are in good agreement with the experimental results in their applicable fields.

The deformation is less than 100%, so the Maxwell model can describe its deformation behavior well. Research on the deformation mechanism of DE materials can effectively improve the understanding of DE materials, in order to design DE materials with a higher performance or specific performance based on the principle. Researchers before 2002 were human, and the electrostrictive behavior of insulating materials was the traditional electrostrictive behavior [35]. In the study of small deformations of DE materials, researchers generally use the Maxwell model to make predictions. The Maxwell stress formula is as follows:

(1) $$P=\varepsilon_{0}\varepsilon_{r}E^{2}$$

where $\varepsilon_{0}$ is the vacuum dielectric constant, $\varepsilon_{r}$ is the relative permittivity, and E is the electrostatic field strength.

Here, we define the strain parameters as follows:

(2) $$\Lambda =\frac{\varepsilon_{0}\varepsilon_{r}}{Y}$$

Among them, Y is the elastic modulus, so for a small deformation, the elastic modulus is considered to be linear and constant, and the strain $S_{Z}$ in the thickness can be expressed as:

(3) $$S_{Z}=-\frac{P}{Y}=-\frac{\varepsilon_{0}\varepsilon_{r}E^{2}}{Y}=-\Lambda E^{2}=-\Lambda {\left(\frac{V}{z}\right)}^{2}$$

Among them, V is the voltage applied on both sides of the DE material, and z is the thickness of the material.

The result of Equation (3) is more consistent with the result of the small deformation of DE material under a DC electric field [36], thus this formula has been widely recognized. People have thought about, analyzed, and predicted electro-induced deformation. At times, this formula is generally used.

Based on the results of the mechanical and dielectric properties of CCTO/PDMS and @CCTO/PDMS, we can know the changes in strain parameters of CCTO/PDMS composites with filling amounts of 5, 10, 15, and 20 wt%, which is 2.5, 1.43, 1.21, and 1.03 times. The strain parameter changes of @CCTO/PDMS composites were 1.93, 1.31, 1.08, and 1.07 times, respectively. We have found that the addition of CCTO can indeed increase its strain parameters, thereby increasing its electro-induced deformation. At the same time, as the amount of CCTO filler increases, its strain parameters decrease, due to fact that the change rate of its elastic modulus is higher than that of the medium, which is the rate of change of the electrical constant.

At the same time, we found that after KH560 is modified, the changes in strain parameters of the composite are lower than those of the unmodified, that is, the enhancement effect of KH560 on mechanical properties is greater than that on dielectric properties.

## 4. Conclusions

In this paper, the dielectric elastomer was prepared by adding different contents of CCTO particles and CCTO particles modified by the silane coupling agent to PDMS, and its mechanical properties, electromechanical properties, etc. were tested. The electro-deformation ability of the two dielectric elastomers was studied separately, the comparative analysis was carried out, and the following conclusions were obtained:

1. The elastic modulus and dielectric constant of the CCTO/PDMS dielectric elastomer are higher than those of the unmodified PDMS, and the conductivity (<10^−7^) and dielectric loss (0.0038) of the dielectric elastomer did not occur significantly. The strain parameter of 5 wt% CCTO/PDMS is increased by 2.5 times compared with PDMS, reaching 33.8%. However, the growth rate of the dielectric constant of the dielectric elastomer is lower than the growth rate of the elastic modulus. As a result, the strain parameter of the dielectric elastomer decreases as the filler content increases. The electromechanical deformation and electromechanical conversion efficiency of the modified material are higher than those of the unmodified PDMS. However, when the amount of filler is too large, the maximum deformation will decrease due to the decrease of the breakdown field strength (14 V/µm).
2. Comparing the dispersion effect of CCTO modified by three silane coupling agents (KH550, KH560, KH570) in PMDS, the dispersion effect of CCTO modified by KH560 is the best. While KH560 improves the dispersion of CCTO, it also improves the dielectric constant and elastic modulus of the dielectric elastomer. When the filler content is 20 wt%, the dielectric constant reaches 6.5 (100 Hz), which is an increase of 150% compared with the unmodified PDMS. However, the effect of KH560 on the modulus of elasticity is greater than the effect on the dielectric constant, resulting in its electromechanical conversion efficiency being lower than CCTO/PDMS. In addition, 5 wt% @CCTO/PDMS has the largest strain parameter, which is 1.93 times higher than that of the unmodified PDMS. The maximum amount of deformation reaches 27.4% at a field strength of 14 V/µm.
3. Through the preparation of CCTO/PDMS composite materials, preparations are made for exploring new research directions and further improving the performance of materials. In the next work, higher performance dielectric elastomers will be pursued.

## Figures and Tables

**Table 1:** List of elements contained in the unmodified CCTO and modified CCTO.

Element	CCTO	KH550-CCTO	KH560-CCTO	KH570-CCTO
C	×	√	√	√
O	×	√	√	√
Si	×	√	√	√
Ca	√	√	√	√
Ti	√	√	√	√
Cu	√	√	√	√

## Data Availability

The data presented in this study are available upon request from the corresponding author. The data are not publicly available due to the laboratory management regulations.

## References

[1] Chidsey C.E.D., Murray R.W. (1986). Electroactive polymers and macromolecular electronics. Science.

[2] Thiele Cathleen D.A.S. (2013). Raghu. Electroactive Polymers and Devices 2013–2018: Forecasts, Technologies, Players.

[3] Sheng J., Chen H., Li B., Chang L. (2013). Temperature dependence of the dielectric constant of acrylic dielectric elastomer. Appl. Phys. A.

[4] Jomaa H., Bchir O.J. (2012). Dual Epoxy Dielectric and Photosensitive Solder Mask Coatings, and Processes of Making Same. U.S. Patent.

[5] Li X.Q., Li W.B., Zhang W.M., Zou H.X., Peng Z.K., Meng G. (2017). Magnetic force induced tristability for dielectric elastomer actuators. Smart Mater. Struct..

[6] Zhao P., Cai Y., Liu C., Ge D., Li B., Chen H. (2020). Study on the bio-inspired electrochromic device enabled via dielectric elastomer actuator. Opt. Mater..

[7] Chiba S. (2014). Dielectric Elastomers.

[8] Pelrine R., Kornbluh R., Pei Q., Joseph J. (2000). High-speed electrically actuated elastomers with strain greater than 100%. Science.

[9] Lai W. (2011). Characteristics of Dielectric Elastomers and Fabrication of Dielectric Elastomer Actuators for Artificial Muscle Applications. Master’s Thesis.

[10] Kofod G. (2001). Dielectric Elastomer Actuators. Chemistry.

[11] Carpi F., Migliore A., Serra G., De Rossi D. (2005). Helical dielectric elastomer actuators. Smart Mater. Struct..

[12] Yang D., Huang S., Ruan M., Li S., Wu Y., Guo W., Zhang L. (2018). Improved electromechanical properties of silicone dielectric elastomer composites by tuning molecular flexibility. Compos. Sci. Technol..

[13] Goulbourne N.C., Mockensturm E.M., Frecker M.I. (2007). Electro-elastomers: Large deformation analysis of silicone membranes. Int. J. Solids Struct..

[14] Zhu J., Stoyanov H., Kofod G., Suo Z. (2010). Large deformation and electromechanical instability of a dielectric elastomer tube actuator. J. Appl. Phys..

[15] Gatti D., Haus H., Matysek M., Frohnapfel B., Tropea C., Schlaak H.F. (2014). The dielectric breakdown limit of silicone dielectric elastomer actuators. Appl. Phys. Lett..

[16] Kumar A., Patra K., Hossain M. (2021). Silicone composites cured under a high electric field: An electromechanical experimental study. Polym. Compos..

[17] Romasanta L.J., Leret P., Casaban L., Hernández M., Miguel A., Fernández J.F., Kenny J.M., Lopez-Manchado M.A., Verdejo R. (2012). Towards materials with enhanced electro-mechanical response: CaCu_3_Ti_4_O_12_-polydimethylsiloxane composites. J. Mater. Chem..

[18] Gallone G., Carpi F., De Rossi D., Levita G., Marchetti A. (2007). Dielectric constant enhancement in a silicone elastomer filled with lead magnesium niobate–lead titanate. Mater. Sci. Eng. C Biomim. Supramol. Syst..

[19] Galantini F., Bianchi S., Castelvetro V., Gallone G. (2013). Functionalized carbon nanotubes as a filler for dielectric elastomer composites with improved actuation performance. Smart Mater. Struct..

[20] Mashingboon C., Thongbai P., Maensiri S., Yamwong T., Seraphin S. (2008). Synthesis and giant dielectric behavior of CaCu_3_Ti_4_O_12_ ceramics prepared by polymerized complex method. Mater. Chem. Phys..

[21] Byeong K.K., Hyung S.L., Lee J.W., Seung E.L., Yong S.C. (2010). Dielectric and grain-boundary characteristics of hot pressed CaCu_3_Ti_4_O_12_. J. Am. Ceram. Soc..

[22] Wang C., Xu F., He M., Ding L., Li S., Wei J. (2018). Castor oil-based polyurethane/silica nanocomposites: Morphology, thermal and mechanical properties. Polym. Compos..

[23] Li H., Wang C., Guo Z., Wang H., Zhang Y., Hong R., Peng Z. (2016). Effects of silane coupling agents on the electrical properties of silica/epoxy nanocomposites[C]//2016 IEEE international conference on dielectrics (ICD). IEEE.

[24] Kang J.S., Yu C.L., Zhang F.A. (2009). Effect of Silane Modified SiO_2_ Particles on Poly (MMA-HEMA) Soap-Free Emulsion Polymerization. https://www.researchgate.net/publication/279553513_Effect_of_silane_modified_SiO2_Particles_on_PolyMMA-HEMA_Soap-free_Emulsion_Polymerization.

[25] Sun Y., Fang X., Ma Z., Xu L., Lu Y., Yu Q., Yuan N., Ding J. (2017). Enhanced UV-light stability of organometal halide perovskite solar cells with interface modification and a UV absorption layer. J. Mater. Chem. C.

[26] Liu G., Chen Y., Gong M., Liu X., Cui Z.K., Pei Q., Gu J., Huang C., Zhuang Q. (2018). Enhanced dielectric performance of PDMS-based three-phase percolative nanocomposite films incorporating a high dielectric constant ceramic and conductive multi-walled carbon nanotubes. J. Mater. Chem. C.

[27] Wang G.L., Zhang Y.Y., Duan L., Ding K.H., Wang Z.F., Zhang M. (2015). Property reinforcement of silicone dielectric elastomers filled with self-prepared calcium copper titanate particles. J. Appl. Polym. Sci..

[28] Zhang Y.Y., Wang G.L., Zhang J., Ding K.H., Wang Z.F., Zhang M. (2019). Preparation and properties of core-shell structured calcium copper titanate@ polyaniline/silicone dielectric elastomer actuators. Polym. Compos..

[29] Wissler M., Mazza E. (2007). Electromechanical coupling in dielectric elastomer actuators. Sens. Actuators A Phys..

[30] Dorfmann A., Ogden R.W. (2005). Nonlinear electroelasticity. Acta Mech..

[31] Suo Z. (2010). Theory of dielectric elastomers. Acta Mech. Solida Sin..

[32] Bustamante (2009). Transversely isotropic non-linear electro-active elastomers. Acta Mech..

[33] Mehnert M., Hossain M., Steinmann P. (2019). Experimental and numerical investigations of the electro-viscoelastic behavior of VHB 4905TM. Eur. J. Mech. A/Solids.

[34] Hossain M. (2020). Modelling the curing process in particle-filled electro-active polymers with a dispersion anisotropy. Contin. Mech. Thermodyn..

[35] Pelrine R.E., Kornbluh R.D., Joseph J.P. (1998). Electrostriction of polymer dielectrics with compliant electrodes as a means of actuation. Sens. Actuators A Phys..

[36] Sahu R.K., Saini A., Ahmad D., Patra K., Szpunar J. (2016). Estimation and validation of maxwell stress of planar dielectric elastomer actuators. J. Mech. Sci. Technol..

